# Intramuscular Haemangioma with Diagnostic Challenge: A Cause for Strange Pain in the Masseter Muscle

**DOI:** 10.1155/2014/285834

**Published:** 2014-06-05

**Authors:** Krithika Chandrasekar Lakshmi, Sathasivasubramanian Sankarapandiyan, Venkata Sai Pulivadula Mohanarangam

**Affiliations:** ^1^Department of Oral Medicine & Radiology, SRM Dental College, Bharathi Salai, Ramapuram, Chennai, Tamil Nadu 600 089, India; ^2^Department of Oral Medicine & Radiology, Faculty of Dental Sciences, Sri Ramachandra University, Porur, Chennai, Tamil Nadu 600 116, India; ^3^Department of Radiology & Imaging Science, Sri Ramachandra University, Porur, Chennai,Tamil Nadu 600 116, India

## Abstract

Intramuscular hemangiomas are unique vascular tumors which are benign in nature, most commonly occurring in the trunk and extremities. When present in head and neck, they most frequently involve the masseter and trapezius muscles, accounting for less than 1% of all hemangiomas. Most of these lesions present with pain and discomfort and some patients may demonstrate progressive enlargement. Due to their infrequency, deep location, and unfamiliar presentation, these lesions are seldom correctly diagnosed clinically. Our report is a clinically misdiagnosed case of a painful soft tissue mass in the right side masseteric region of a 23-year-old female patient, confirmed as intramuscular hemangioma based on imaging studies and histopathologic examination, treated by surgical excision which had no recurrence after a 3-year followup.

## 1. Introduction

Hemangiomas are vascular neoplasm, constituting 7% of all benign tumors [1]. These are tumors of infancy, occurring most frequently on cutaneous and mucosal surfaces. Such tumors occurring in the skeletal muscles of head and neck region are rare. Intramuscular hemangiomas make up 0.8% of all hemangiomas [2]. Approximately 14% of intramuscular hemangiomas are manifested in the head and neck, with masseter muscle representing the most common site of involvement [3]. Due to their rarity, deep seated location, and bizarre clinical presentation, intramuscular hemangioma should be considered in the differential diagnosis of nonspecific soft tissue swelling with strange pain.

## 2. Case Report

**Figure 1:**
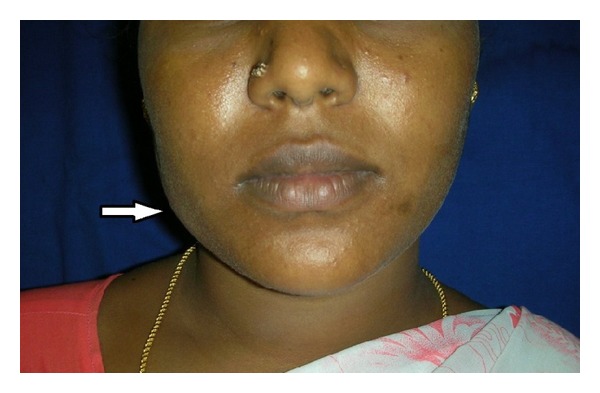
A 23-year-old female patient, presenting with a mild diffuse swelling on the right side of the face.

A 23-year-old lady reported to the Department of Oral Medicine & Radiology, Sri Ramachandra University and Research Institute, with a complaint of swelling in the right side of the face for the past 6 months. She had incidentally noticed the swelling which remained of the same size for 3 months, after which it became more bulging. Over the last 3 months she experienced intermittent pricking type of pain. Medical history was unremarkable. Clinical examination revealed a diffuse swelling in the right mandibular body region measuring about 2 × 2 cm in dimension, which was warm, tender, and soft in consistency. The swelling was mobile in the horizontal direction and showed restricted mobility in the vertical plane. Overlying skin was pinchable (Figure 1). On clenching the swelling became more prominent. Intraoral examination revealed no abnormality. A clinical diagnosis of buccal node lymphadenitis and a differential diagnosis of soft tissue abscess, masseteric hypertrophy, accessory parotid tumor, and lymphovascular tumor were considered and the necessary diagnostic workup was done.

**Figure 2:**
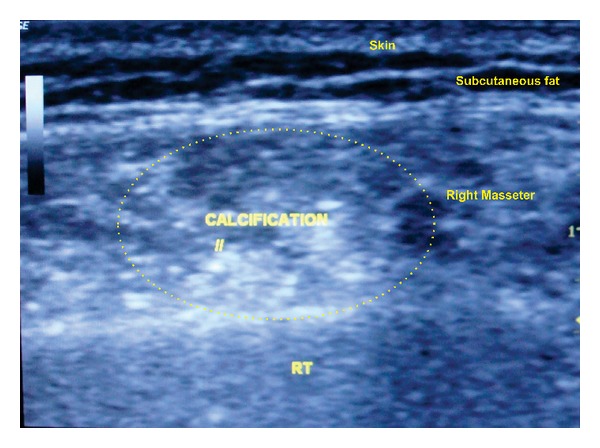
Grey scale ultrasound image revealing a mixed echo lesion with a phlebolith in the right masseter.

**Figure 3:**
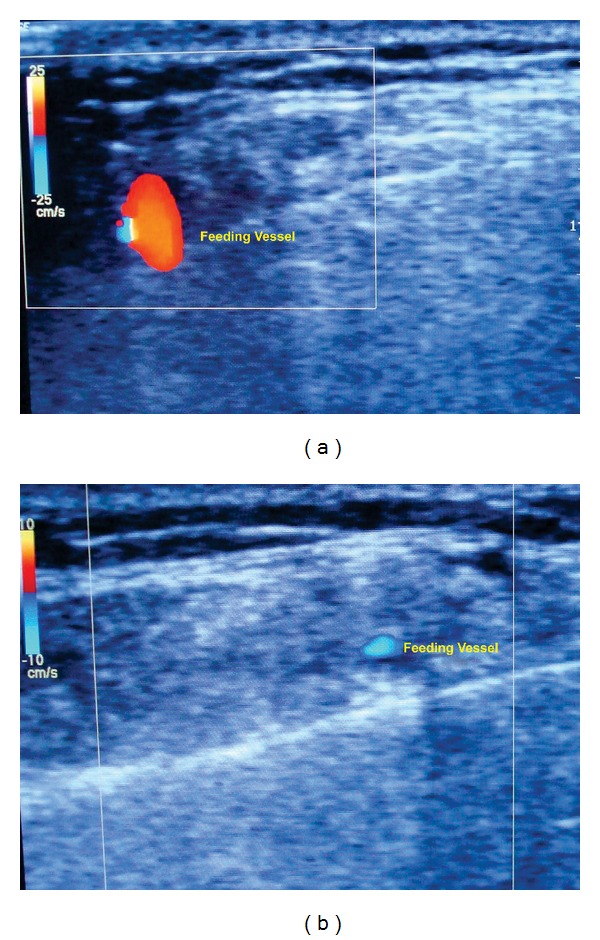
(a, b) Colour Doppler sonography showing internal vascularity.

**Figure 4:**
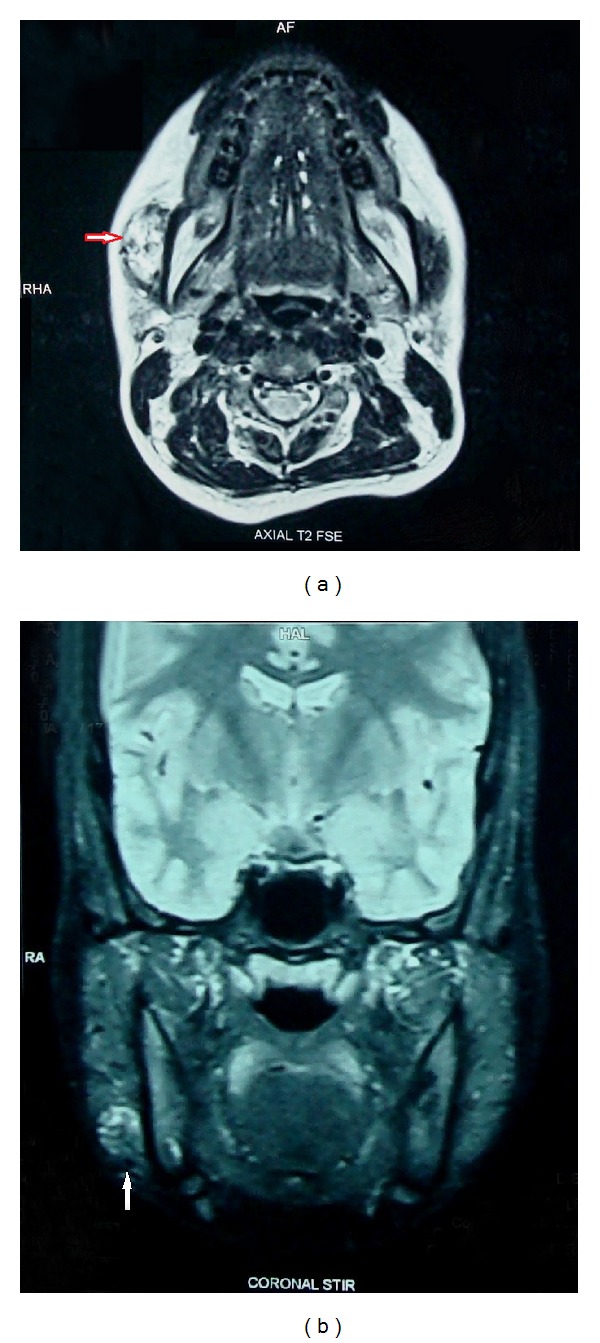
(a) T2 weighted axial view, MRI showing mixed hypo and hyperintense mass, along the anteroinferior aspect of the right masseter. (b) T2 weighted, fat suppressed coronal view of the lesion in right masseter.

Routine hematological investigations were within normal limits. Posteroanterior view of mandible showed no pathology; ultrasonography showed a 2.2 × 0.6 cm mixed echoic lesion within the right side masseter muscle with a speck of calcification (Figure 2). Left masseter appeared normal. Colour Doppler ultrasound showed dilated vascular channels with good flow (Figures 3(a) and 3(b)). There were no interarterial/venous communications. MRI showed a small well defined T2 mixed hypo and hyperintense mass signal, space occupying lesion (SOL), along the anteroinferior aspect of the right masseter, measuring 2.1 × 2.5 × 1.8 cm in dimension in the axial view (Figure 4(a)) and fat suppressed T2 coronal view (Figure 4(b)).

**Figure 5:**
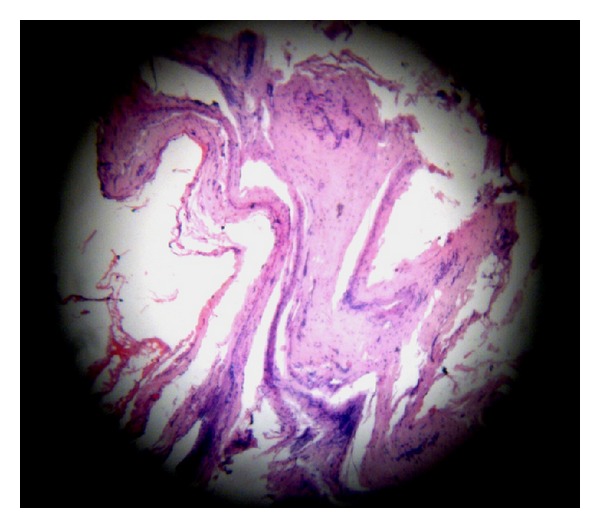
H&E histological section showing fibrofatty tissue and fragments of muscle with several thick walled and thin walled vessels and areas of hemorrhage with congestion.

**Figure 6:**
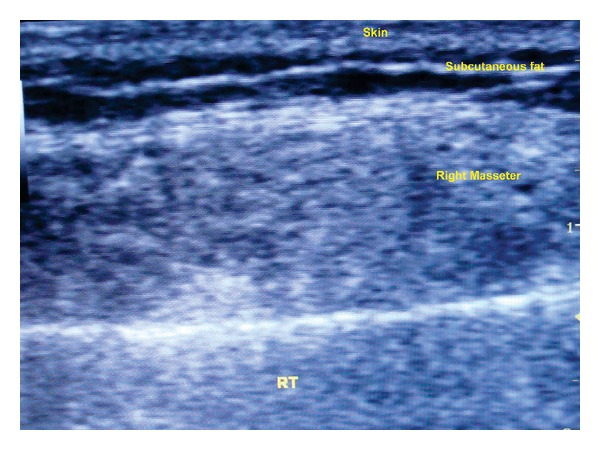
Grey scale sonography showing no evidence of the lesion after a 3-year followup.

Under general anesthesia, preauricular skin incision was made, dissecting lateral to the parotid gland, and skin flaps were raised. Within the masseter a small bulging mass, measuring about 2 × 2.5 cm in dimension, was evident. The branches of the facial nerve were preserved. The external carotid artery was looped and proximal vascular control was achieved; small feeding vessels were individually ligated and blood loss during the procedure was minimal. The mass was completely removed with a margin of normal surrounding muscle to prevent recurrence. Primary closure was done. Postsurgically she was prescribed with antibiotics and analgesics for 5 days. There was mild postoperative facial edema, which subsided within twenty days with no evidence of pain and significant cosmetic problem. Histological examination revealed fibrofatty tissue and fragments of muscle with several thick walled and thin walled vessels with occasional nerves. There were also areas of hemorrhage and congestion, suggestive of a venous hemangioma (Figure 5). After a 3-year followup patient was asymptomatic and ultrasonography revealed no evidence of lesion (Figure 6).

## 3. Discussion

Hemangiomas are benign vascular neoplasms or hamartomas, which are indigenous to the site of origin. Intramuscular hemangiomas are very rare with masseter muscle accounting for 5% of all intramuscular hemangiomas; other frequently involved muscles are trapezius, extraocular muscles, sternocleidomastoid, and temporalis. Their growth may be accelerated with a growth spurt or trauma and tend to enlarge slowly. They can spontaneously regress. Malignant transformation is rare. A sudden increase in size on taking oral contraceptive pills has also been reported [2]. They are usually detected early. These tumors generally present as enlarging soft tissue masses with or without pain. Signs and symptoms may suggest the vascular nature. 90% of the cases occur before the age of 30 years [3].

Most hemangiomas can be diagnosed on clinical examination and do not require any investigation or any treatment as they tend to subside spontaneously. However, imaging is needed in cases of deep hemangioma with normal overlying skin or in cases of clinically atypical soft-tissue masses. When imaging is used, it is important to choose the modality based on the specific lesion and clinical situation. Conventional radiographs help in identifying phlebolith and calcifications, but they may not be specific. Ultrasonography and magnetic resonance imaging (MRI) are the commonly used modalities of choice. In intramuscular hemangiomas, Colour Doppler sonography is exclusively useful to demonstrate the vascular structures in and around the muscle and to evaluate the pathological changes like fibrosis and to detect calcifications. In our case presence of calcification was not evident on plain radiography and MRI, but sonography demonstrated its presence. Hemangioma can be distinguished from other soft tissue lesions by the features of abundant vascularity and high blood flow velocity. Colour Doppler signal in a well-defined hypoechoic mass with heterogeneous echotexture should raise the possibility of hemangioma [4]. Hemangioma with arterial flow can be distinguished from arteriovenous malformations (AVM) by the presence of solid parenchymal tissue and interarterial communications. MRI aids in discerning and delineating deep situated and large intramuscular hemangioma, and it gives the best diagnostic information. The MRI findings of an intramuscular hemangioma consist of an intermediate signal on T1 weighted images and an intense signal on T2 weighted images [5], but it should be noted that not all intramuscular hemangiomas will give a high-intensity signal on T2 weighted MRI [6]. If pulsations, bruits, or thrills are evident on clinical examination, arteriography is indicated to identify large vessel communications [7].

On pathologic analysis, vascular lesions can be classified as capillary, cavernous, venous, and arteriovenous malformations depending on the predominant anomalous vascular channels.

In our case, imaging studies revealed that the lesion is present within the masseter muscle and the differential diagnosis includes masseteric hypertrophy which can be unilateral or bilateral and mostly asymptomatic. Etiology includes defective occlusion, temporomandibular joint disorder, congenital and functional hypertrophies, and emotional disorders. Increased masseteric bulk in sonography is diagnostic. Soft tissue abscesses are mostly due to bacterial infections and appear as focal hypoechoic area with echogenic debris, pus, and occasionally gas. Malformations can be grouped as either high-flow (arteriovenous) or low-flow (capillary, cavernous, and venous) vascular lesions. Flow characteristics are best demonstrated by Doppler sonography. Lymphatic malformations are present from birth and are usually detected before the age of 2 years. They generally appear cystic with thick or thin septae with interspersed solid areas on sonographic examination.

The treatment of choice is total excision. Surgical excision is associated with 9–28% recurrence rate [8], because of the infiltrative growth pattern. Sclerotherapy has a role in the management of intramuscular haemangioma when excision is not possible.

Intramuscular hemangioma should be considered in the differential diagnosis, whenever a soft tissue lesion with pain in a skeletal muscle of a young adult is encountered. Sonography and MRI are excellent diagnostic aids in such lesions.
